# Biomechanical Outcomes of Surgically Repaired TFCC Palmer Type 1B Tears: A Systematic Review of Cadaver Studies

**DOI:** 10.1177/15589447221105546

**Published:** 2022-07-09

**Authors:** Claire Elisabeth Arnolda Koeyvoets, Joris Sebastiaan Teunissen, Reinier Feitz, Steven Hovius, Elisabeth Hagert, Egberta Petronella Adriana van der Heijden

**Affiliations:** 1 Radboud University Medical Center (Radboudumc), Nijmegen, The Netherlands; 2 Xpert Clinics, Amsterdam, The Netherlands; 3 Xpert Clinics, Rotterdam, The Netherlands; 4 Karolinska Institutet, Stockholm, Sweden; 5 H.M. Queen Sophia Hospital, Stockholm, Sweden; 6 Sophiahemmet University, Stockholm, Sweden; 7 Jeroen Bosch Hospital, Hertogenbosch, The Netherlands

**Keywords:** cadaver studies, DRUJ instability, systematic review, TFCC, triangular fibrocartilage complex repair

## Abstract

**Background:**

Palmer type 1B triangular fibrocartilage complex (TFCC) tears are a common cause of distal radioulnar joint (DRUJ) instability. Unfortunately, the best surgical technique for TFCC reinsertion is still unknown, and up to a quarter of patients report instability after repair. The purpose of this systematic review of cadaver studies was to compare the biomechanical outcomes of different surgical techniques used for Palmer 1B TFCC tears.

**Methods:**

A systemic review of all cadaver studies published before January 2022 was performed using the PubMed and EMBASE databases. Only cadaver studies on reinsertion techniques for Palmer type 1B lesions were included. Biochemical outcome parameters evaluated were stability of the DRUJ and strength of the repair.

**Results:**

A total of 248 articles were identified. Five articles fulfilled the inclusion criteria. Four different surgical techniques were identified. In 3 studies, transosseous tunnel repair was tested and resulted in the most stable DRUJ and strongest TFCC repair compared with the suture anchor repair, the peripheral capsular repair, and the outside-in repair.

**Conclusions:**

These results suggest that the transosseous tunnel repair might be a good technique for restoring DRUJ stability. However, more cadaver studies are needed to identify the most optimal technique.

## Introduction

Palmer type 1B triangular fibrocartilage complex (TFCC) lesions occur relatively frequently and are often caused by a fall on an outstretched, pronated hand or by a forced traction and twisting motion of the wrist.^1,2^ These lesions might lead to instability of the distal radioulnar joint (DRUJ), complaints of ulnar-sided wrist pain, impaired function, and decreased grip strength.^3,4^

In the case of persisting symptomatic DRUJ instability despite nonoperative treatment, surgical repair is the next choice. If the TFCC is still of sufficient quality and firm, reattachment is preferred due to the good vascularization and healing tendency of the periphery of the TFCC.^5,6^

Several techniques of reattachment have been described, yet no technique has proven to be superior. Two recent systematic reviews found comparable results for arthroscopic and open approaches regarding pain, function, range of motion, and complications.^7,8^ A recent study by Feitz et al showed that after 5 years, 83% of patients treated by an open TFCC reinsertion reported a minimal clinical improvement. The other 17%, however, did not assess the result as clinically relevant.^9^ In addition, clinical studies reported recurrence of DRUJ instability after successful TFCC repair in 4% to 29% of patients.^8,10^ Moreover, a long-term study even reported the development of osteoarthritis after 15 to 20 years.^11^ Unfortunately, the correlation between clinical results and the surgical technique of TFCC repair is not known. One of the reasons might be that mainly approaches (open vs arthroscopic) have been compared instead of different reattachment techniques by one approach.

Whereas clinical studies focus on patient-related outcome measures, pain, motion, and strength measurements, cadaver studies can evaluate the effect of different techniques on the recovery of DRUJ stability by assessing biomechanical outcomes such as the amount of ulnar translation relative to the radius. In cadavers, translation movements between the ulna and the radius can be measured objectively and quantified, giving a more accurate indication of possible DRUJ translation than imaging techniques or physical examination.^12,13^ For example, testing DRUJ stability in patients is subjective and has low interrater reliability.^12,14^ Also, various computed tomographic (CT) methods for evaluating DRUJ instability lack accuracy and still underestimate and overestimate the extent of subluxation.^15-18^

Until now, no study has summarized the outcomes of the biomechanical cadaver studies. This results in a knowledge gap regarding the technique that restores stability to the greatest extent.

We aimed to systematically review the cadaver studies on the biomechanical outcomes of different surgical techniques used for Palmer 1B TFCC tears by evaluating the restoration of DRUJ stability and strength of the repair.

## Materials and Methods

### Databases and Search

This systematic review was conducted using the Preferred Reporting Items for Systematic Reviews and Meta-Analyses guidelines.^19^ The electronic databases MEDLINE (PubMed) and Embase (Elsevier) were searched for eligible articles published before January 2022. The following MeSH terms and keywords were used: “distal radio-ulnar joint,” “triangular fibrocartilage*,” “anatom*,” “biomechanic*,” and “cadaver*,” including abbreviations and alternative ways for orthography. There were no restrictions on publication date. The complete search strategies are included in Supplemental Table 1.

### Study Selection and Eligibility Criteria

The inclusion of eligible articles was conducted by 2 independent reviewers (CK, JT), and disagreement was resolved with consensus between the 2 raters and senior author (EH).

Articles were eligible for inclusion if they: (1) concern cadaver studies only on Palmer type 1B TFCC lesions; (2) evaluate a surgical technique for reinsertion of the TFCC; (3) provided at least one of the following biomechanical outcomes: translation of the ulna relative to the radius and strength of the repair; and (4) were written in English.

Exclusion criteria were: (1) clinical commentary, research letter, editorial note, finite element models, articles concerning imaging, or measurement tools; (2) articles concerning only the description of surgical techniques for reinsertion of the TFCC without testing biomechanical outcomes; (3) articles about structures in the upper extremity other than the TFCC; and (4) reviews.

All references were screened for eligibility on title and abstract, and if potentially eligible for inclusion, the full-text versions were obtained.

### Quality Assessment

The methodological validity of the included articles was assessed by 2 authors (CK, JT), using the Quality Appraisal for Cadaveric Studies (QUACS).^20^ This scale uses a 13-item checklist including a clear description of: (1) the study aim; (2) the sample; (3) the condition of the specimen; (4) the study protocol; (5) the expertise level of the dissecting researchers; (6) interobserver reliability; (7) results; (8) statistic tools; (9) the consistency of findings; (10) photographs of the observations; (11) results in relation to current evidence; (12) clinical relevance; and (13) study limitations. Each item can be scored as “yes” or “no” (1 or 0 points), with an exact description of when to score “yes.” An overall score will be calculated and expressed as a percentage. If a discrepancy of more than 1 point between the assessments existed, this was discussed with the senior investigator (EH) to reach a consensus.

### Data Extraction

In addition to the quality assessment, the following data were extracted from each of the included studies: first author, year and journal of publication, cadaver sample characteristics, surgical techniques used, testing protocols used (force measurements, wrist positions), and outcome measures: recovery of DRUJ stability and strength of the TFCC repair.

### Outcome Measures

The primary outcome was restoration of DRUJ stability measured by the preoperative and postoperative amount of volar and dorsal translation of the radius relative to the ulna. The amount of translation relative to the uninjured wrist was calculated and expressed as a percentage of eliminated translation (P_ET_) postoperatively (Supplemental Table 2). The secondary outcome was strength of the TFCC repair, measured by load to failure (in Newton), and mechanism of failure of the repair. Measurements of translation and strength were determined in different wrist positions: neutral, or full pronation and full supination. The different wrist positions used are reported per study.

The results regarding stability and strength will be tabulated, and the best technique independent of the parameter tested will be identified for each included study.

## Results

### Search and Selection

**Figure 1.**
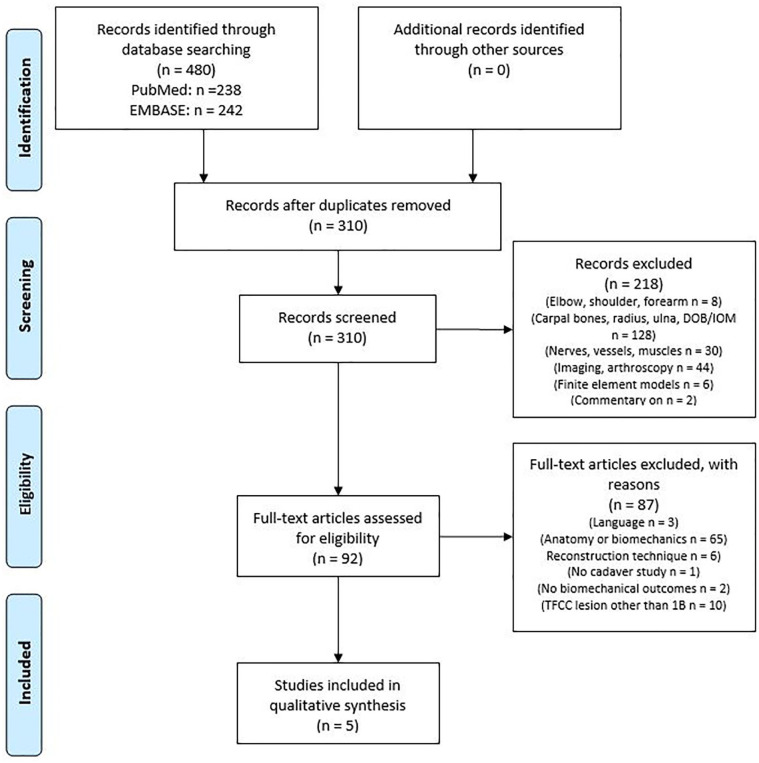
Preferred Reporting Items for Systematic Reviews and Meta-Analyses flow diagram of inclusion and exclusion. *Note*. IOM = interosseous membrane; DOB = distal oblique band.

After applying the inclusion and exclusion criteria, 5 of the 248 initially identified articles were included, all published between 2003 and 2021 (Figure 1 and Table 1).

**Table 1.** Study Characteristics.

Article	Journal of publication	Quality assessment	Surgical techniques	No. of arms	Age and sex (mean age, M/F)	Quality mentioned	Wrist position
Desai et al.^21^	*American Journal of Hand Surgery*	77%	Outside-in repair Suture anchor repair	12 (matched-pairs)	—	Yes	Neutral
Gutiérrez-Monclus et al.^22^	*Science Progress*, SAGE journals	77%	Suture anchor repair Transosseous tunnel repair	12 (unmatched)	53 y 6/6	Yes	Pronation, supination
Johnson et al.^23^	*HAND*	62%	Peripheral capsular repair Transosseous tunnel repair	16 (matched-pairs)	67 y, 6/2	Yes	Pronation, supination
Ma et al.^24^	*The Journal of Arthroscopic and Related Surgery*	85%	Suture anchor repair Transosseous tunnel repair	12 (matched-pairs)	—	Yes	Neutral
Yao^25^	*American Journal of Hand Surgery*	62%	Outside-in repair Peripheral capsular repair	20 (matched-pairs)	—	Yes	Neutral

*Note*. Specifications of surgical techniques are in Supplemental Table 4.

### Quality Assessment

The mean QUACS score of the included studies was 73% (range, 62%- 85%), indicating a moderate quality (Table 1). Overall, the main weaknesses were missing data concerning sex, age, and quality of the cadaveric arms and the expertise level of the dissecting researcher (Supplemental Table 3).

### Study Characteristics

The characteristics of the included studies are shown in Table 1. All studies created a Palmer type 1B TFCC tear. A total of 72 cadaver arms (range, 12-20) were included in this review. Four of the included studies used matched-pairs of fresh-frozen cadaveric forearms.^21,23-25^ The fifth study by Gutiérrez-Monclus et al^22^ did not describe the preparation of their cadavers and used independent wrists. All included studies used wrists without pre-existing pathology.

**Figure 2.**
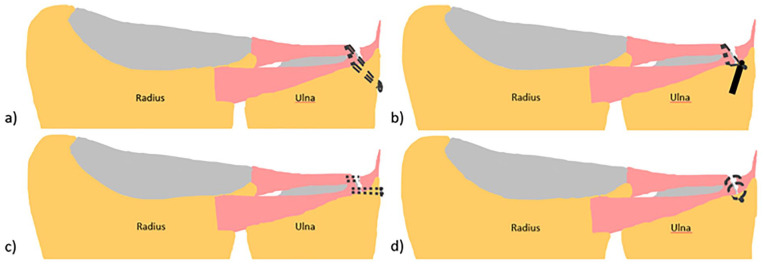
Illustrations of the different techniques for reinsertion of the triangular fibrocartilage complex: (a) illustration of transosseous tunnel repair, (b) illustration of suture anchor repair, (c) illustration of peripheral capsular repair, and (d) illustration of inside-out repair. The articular disk is not depicted to give a better overview of the surgical techniques.

Four different surgical techniques of TFCC reinsertion were used: the transosseous tunnel repair, the suture anchor repair, the peripheral capsular repair, and the outside-in repair (Figure 2).^21-25^

Johnson et al^23^ compared the transosseous tunnel repair with the peripheral capsular repair. For the arthroscopic transosseous tunnel repair, a 3-mm intra-articular hole was drilled just proximal from the ulnar neck to the fovea. A suture lasso was fed through the tunnel into the joint, through the TFCC, and back through the tunnel with the assistance of a 2-0 FiberWire. The suture was secured 1 cm proximal to the ulnar tunnel with a 2-mm Push Lock anchor. For the open peripheral capsular repair, three 2-0 polydioxanone sutures (PDS) were used as described by Ruch and Papadonikolakis.^26^ The sutures were placed in a horizontal mattress fashion, using meniscal needles, to secure the TFCC to the capsule just volar from the extensor carpi ulnaris subsheath (Supplemental Table 4).

Like Johnson et al, Ma et al^24^ applied the transosseous tunnel repair but compared this technique with the suture anchor technique. Ma et al drilled 2 transosseous tunnels instead of one, just proximal to the base of the ulnar styloid to the fovea. They used no. 2 braided USP core sutures to pass through one of the tunnels, the TFCC, and back through the other tunnel assisted by a looped nylon 3-0 suture. Both ends of the suture were tied together. For the suture anchor technique, a 3.5-mm suture anchor with ultrabraid sutures was placed at the fovea, and both ends of the suture were passed through the TFCC, one through the palmar ligament and one through the dorsal ligament. The suture knots were placed outside the joint and tightened with the wrist in a neutral position (Supplemental Table 4).

Gutiérrez-Monclus et al^22^ compared the transosseous tunnel repair with the suture anchor repair. For the transosseous tunnel repair, same as in the study by Ma et al, 2 transosseous tunnels were drilled. A FiberWire 2.0 suture was passed through the bone tunnel, through the TFCC, and back through the other bone tunnel with the assistance of hypodermic needle no. 18. Four knots were tied with both ends of the suture. For the suture anchor technique, a 2.7-mm Anchor Corkscrew and 2.0 FiberWire were used. The anchor was placed in the fovea, and both ends of the suture were passed through the TFCC (Supplemental Table 4).

Desai et al^21^ compared the suture anchor technique with the outside-in TFCC repair. For the suture anchor repair, 2-0 fiber wires were used, and a mini-pushlock suture anchor was placed into the fovea, as described by Geissler.^27^ For the outside-in repair, two 2-0 PDS sutures were applied in a vertical mattress fashion as described by Whipple and Geissler^28^ (Supplemental Table 4).

Finally, Yao^25^ compared the outside-in repair with the peripheral capsular repair. The same technique as Desai et al was used for the outside-in repair. The peripheral capsular repair was performed by the FasT-Fix system—deploying 2 pre-tied 0 Ti-Cron sutures in the TFCC and capsule both volar and dorsal in the TFCC (Supplemental Table 4).

### DRUJ Stability Tested by Amount of Dorsopalmar Translation

Dorsopalmar translation was measured by Ma et al^24^ and Johnson et al^23^ by applying a fixed mechanical force in the dorsal and volar direction. We calculated the P_ET_ for Johnson et al^23^ from the results mentioned in their study (Table 2). Ma et al^24^ already noted the P_ET_ values.

**Table 2.** Outcomes Per Study for DRUJ Stability and Strength, With Identification of the Best Technique for Each Study.

Study	Surgical techniques	DRUJ stability (P_ET_)	Strength of TFCC repair (N / no. of cycles)	Best repair (independent of the parameter tested)*
Desai et al.^21^	Suture anchor repair Outside-in repair		10 ± 3 N 2 ± 1 N	Suture anchor repair
Gutiérrez-Monclus et al.^22^	Transosseous tunnel repair Suture anchor repair		41.7 cycles 28.3 cycles	Transosseous tunnel repair
Johnson et al.^23^	Transosseous tunnel repair Peripheral capsular repair	P_ET_ 77% P_ET_ 12%		Transosseous tunnel repair
Ma et al.^24^	Transosseous tunnel repair Suture anchor repair	P_ET_ 172% P_ET_ 64%		Transosseous tunnel repair
Yao^25^	Peripheral capsular repair Outside-in repair		3.7 N 2.4 N	Peripheral capsular repair

*Note.* DRUJ = distal radioulnar Joint; TFCC = triangular fibrocartilage complex.

* *P* < .05.

**Figure 3.**
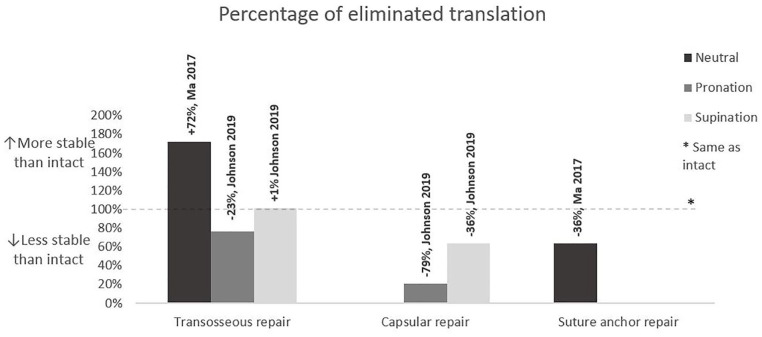
Percentage of eliminated translation (P_ET_) for different surgical techniques. A P_ET_ above 100% indicates a more stable wrist after reconstruction and below 100% a less stable wrist after reconstruction. The corresponding study is displayed above each bar.

Ma et al^24^ compared the transosseous tunnel repair with the suture anchor repair in a neutral wrist position. Both types of repair reduced translation in the DRUJ, but the transosseous tunnel repair reached a more stable DRUJ compared with the suture anchor repair, with a median P_ET_ of 172% and 64%, respectively (*P* = .043) (Figure 3 and Table 2).

Johnson et al^23^ compared the transosseous tunnel repair with the peripheral capsular repair in pronation and supination. They also found decreased translation after both reinsertion techniques, but more after the transosseous tunnel repair. The P_ET_ for the transosseous tunnel repair and the peripheral capsular repair in pronation was 77% versus 12%, respectively (*P* = .01), and in supination was 101% versus 64%, respectively (*P* < .01) (Figure 3 and Table 2).

### Strength of TFCC Repair Tested by Load to Failure and Mechanism of Failure

Three studies assessed the strength of TFCC repair for all 4 reinsertion techniques. Desai et al^21^ and Yao^25^ used the same study protocol. Both indicated the load to failure as the amount of force necessary to create a 2-mm-wide gap across the repair site in a neutral wrist position. In contrast, Gutiérrez-Monclus et al^22^ determined load to failure as the number of pronation and supination cycles necessary to create a 2-mm-wide gap across the repair site. The same classification for mechanism of failure was used by all 3 studies.

**Figure 4.**
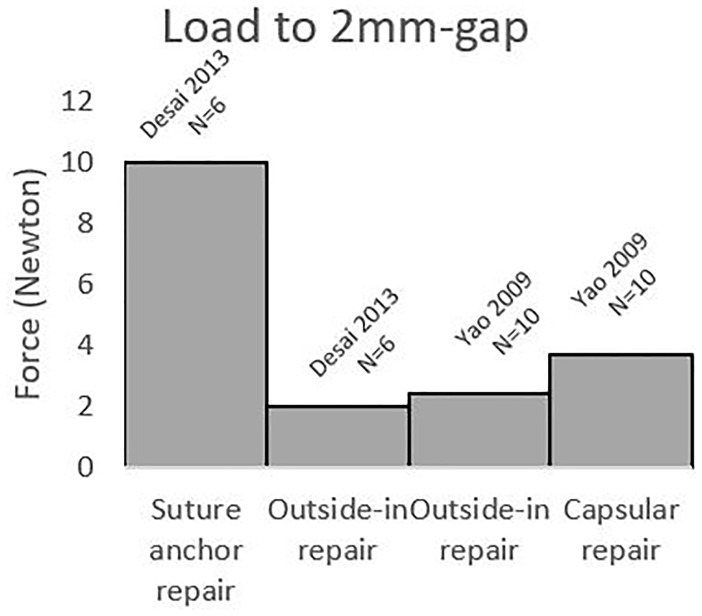
Maximum amount of load before a gap of 2 mm formed across the repair site for the 3 different surgical techniques.

Desai et al^21^ tested the suture anchor repair and the outside-in repair. The load necessary for creation of the 2-mm-wide gap for the suture anchor repair was 10 ± 3 N and 2 ± 1 N for the outside-in repair. This indicates that the suture anchor repair is stronger (*P* < .05) (Figure 4 and Table 2).

Yao^25^ compared the peripheral capsular repair with the outside-in repair. They found favorable results for the peripheral capsular repair, with an average load to failure of 3.7 N, compared with 2.4 N for the outside-in repair (*P* < .05) (Figure 4 and Table 2).

Gutiérrez-Monclus et al^22^ compared the transosseous tunnel repair with the suture anchor repair. They found a mean of 41.7 pronation and supination cycles necessary for failure of the transosseous tunnel repair and only 28.3 cycles before failure of the suture anchor repair. This indicates that the transosseous tunnel repair is stronger (*P* = .025) (Figure 4 and Table 2).

**Figure 5.**
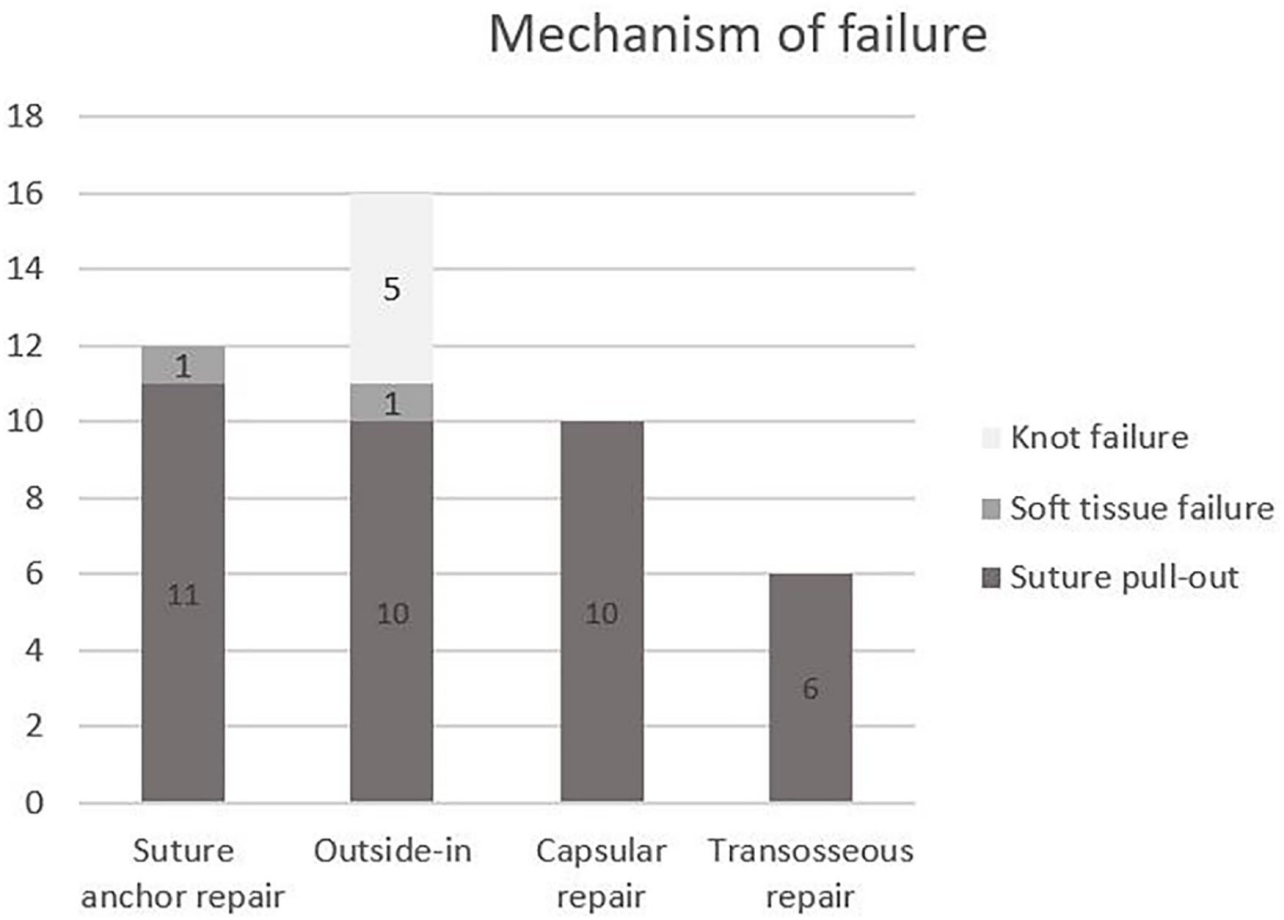
Mechanism of failure of different surgical techniques of triangular fibrocartilage complex reinsertion.

The predominant cause of failure was suture pullout of the soft tissue of the TFCC. In the study by Desai et al,^21^ suture pullout occurred in 5 of 6 cadavers after the suture anchor repair and in 3 of 6 cadavers after the outside-in repair. After the outside-in repair by Yao,^25^ 7 of 10 cadavers had suture pullout, and this was the mechanism of failure for all cadavers after the peripheral capsular repair. In the study by Gutiérrez-Monclus et al,^22^ suture pullout was the only occurring mechanism of failure after both reinsertion techniques. Knot failure only occurred after the traditional outside-in repair in the study by Yao^25^ (Figure 5).

## Discussion

Distal radioulnar joint instability is most often caused by a TFCC Palmer type 1B lesion.^4,29^ Till now, clinical studies have not revealed the best surgical technique to repair this lesion. Cadaver studies have been performed to evaluate the biomechanical properties of different TFCC repair techniques, but the results of these studies have not been summarized yet. Initially, we aimed to perform a meta-analysis of cadaver studies comparing different repair techniques of TFCC Palmer type 1B tears. However, this was not feasible due to heterogeneity of the studies. The transosseous tunnel repair appeared to be the best in 3 studies, in which this technique was compared with the suture anchor repair or the peripheral capsular repair. The outside-in appeared to be the worst repair.

Five cadaver studies could be included in our systematic review in which 4 different reattachment techniques of type 1B TFCC lesions were used: the transosseous tunnel repair, the suture anchor, the peripheral capsular repair, and the outside-in repair. The techniques studied are comparable to the reinsertion techniques most often used clinically.^7-9^ In each cadaver study, 2 different surgical techniques were compared. The primary outcome was restoration of DRUJ stability measured by the preoperative and postoperative amount of volar and dorsal translation of the radius relative to the ulna in different wrist positions. Distal radioulnar joint stability was tested prior to the creation of the TFCC 1B lesion, after the creation of the TFCC 1B lesion, and after the repair of the TFCC 1B lesion. For comparison reasons, we expressed stability as the percentage of eliminated translation after TFCC repair (P_ET_).^24^ This percentage expresses how well the repair recreates stability, that is, how well the amount of translation of the wrist with an intact TFCC was obtained. By expressing postoperative DRUJ instability as a percentage of the uninjured wrist, a correction for pre-existing differences in joint stability and tissue quality between wrists has been performed. The transosseous tunnel repair was found to give more DRUJ stability compared with the suture anchor technique and the peripheral capsular repair.^23,24^

A possible explanation for this finding might be that the oblique course of sutures in the transosseous tunnel technique exerts an oblique traction force enabling firmer reinsertion of the TFCC in contrast to the transverse traction force of sutures in the other 2 techniques. Another explanation might be the possibility to pull the avulsed TFCC more tightly into the fovea when the sutures are tunneled through to the other side of the ulna, resulting in less laxity of the reinserted TFCC.

The strength of the TFCC repair was the second outcome of interest and was tested in 3 independent studies. In correspondence with our findings concerning the recovery of DRUJ stability, the transosseous tunnel repair appeared to give the strongest repair in comparison with the suture anchor repair, which in turn appeared stronger than the capsular peripheral repair.^21,22,25^ The most common reason for failure in all techniques was suture pullout from the soft tissue of the TFCC.^21,22,25^ Knot failure only occurred after the outside-in repair, with 2-0 PDS sutures.^25^

During the conduct of this systematic review, we encountered several limitations that were related to the available literature. The included studies used slightly different surgical techniques for TFCC reinsertions. Moreover, none of the studies tested the techniques in all wrist positions: neutral, pronation, and supination. Because of lack of data concerning age, sex, cadaver preparation, and heterogeneity in methodology, data could not be pooled, and no statistical analysis could be performed. We needed to adhere to the comparisons between techniques made within a single study and could not draw an overall conclusion. Another limitation is that cadaver studies do not necessarily translate directly to the clinic. First, cadaveric wrists are usually from elderly patients. This might result in poorer or more inconsistent tissue quality. However, within a study, the quality of both study groups was similar as arms were randomly assigned to different reinsertion techniques. In addition, each arm served as its own control. Second, with cadavers, it is difficult to simulate exactly the same forces that the wrist has to endure in real life. In cadaver studies, a worst-case scenario was created by applying as much load over the repair site as was necessary to destroy the repair, a technique also applied in research on strength of tendon repairs.^30,31^ Third, in cadavers there is no wound healing process that might influence the final stability and strength of a repair.

Despite these limitations, cadaver studies also have strengths, particularly in anatomical and biomechanical research. With cadavers, it is possible to focus on the techniques themselves, without the interference of factors, such as scar tissue formation, complications, and patient-related factors. Stability of the DRUJ and strength of the repair are important parameters in TFCC reinsertion, which cannot be tested objectively in patients. Physical examination of stability is subjective, and imaging of the DRUJ using CT is still missing some accuracy.^12,15^ With cadaver studies, objective measurements that can identify the best technique in restoring DRUJ stability can take place . Because of these strengths, more cadaver studies should be performed, with standardized protocols, to evaluate biomechanical differences between the included techniques. Only with those protocols, we will be able to draw firm conclusions.

Two recent systematic reviews on clinical studies using the same 4 techniques as evaluated in the cadaver studies found comparable results for the different techniques. However, they combined the techniques and only focused on identifying any differences between an open and an arthroscopic approach. Till now, most other studies compared open and arthroscopic approaches for TFCC repair. It might be that the technique used for TFCC repair is of more importance than the approach itself. The advantage of cadaver studies is that both different techniques and approaches can be compared. Also, different suture materials could relatively simply be compared. After identifying the technique giving the best DRUJ stability and strongest TFCC repair, this technique could be further analyzed in the clinic.

## Supplemental Material

sj-docx-1-han-10.1177_15589447221105546 – Supplemental material for Biomechanical Outcomes of Surgically Repaired TFCC Palmer Type 1B Tears: A Systematic Review of Cadaver Studies Click here for additional data file. Supplemental material, sj-docx-1-han-10.1177_15589447221105546 for Biomechanical Outcomes of Surgically Repaired TFCC Palmer Type 1B Tears: A Systematic Review of Cadaver Studies by Claire Elisabeth Arnolda Koeyvoets, Joris Sebastiaan Teunissen, Reinier Feitz, Steven Hovius, Elisabeth Hagert and Egberta Petronella Adriana van der Heijden in HAND
