# Supplementary material for: Biomechanical Outcomes of Surgically Repaired TFCC Palmer Type 1B Tears: A Systematic Review of Cadaver Studies
Source: Hand (N Y). 2022 Jul 9;18(8):1258–66. doi: 10.1177/15589447221105546 (PMC10617480; doi:10.1177/15589447221105546)
Supplement: sj-docx-1-han-10.1177_15589447221105546 – Supplemental material for Biomechanical Outcomes of Surgically Repaired TFCC Palmer Type 1B Tears: A Systematic Review of Cadaver Studies [file sj-docx-1-han-10.1177_15589447221105546.docx]

**Supplements**

*Supplement Table 1. Search Strategy*

Search Pubmed: 25-01-2022

| 1 | ((distal radio-ulnar joint[Title/Abstract]) OR (distal radioulnar joint[Title/Abstract])) OR (DRUJ[Title/Abstract]) | 1853 |
| --- | --- | --- |
| 2 | (("Triangular Fibrocartilage"[MeSH]) OR (triangular fibrocartilage*[Title/Abstract])) OR (TFCC[Title/Abstract]) | 1384 |
| 3 | #1 OR #2 | 2720 |
| 4 | ("Anatomy"[MeSH]) OR (Anatom*[Title/Abstract]) | 781412 |
| 5 | ("Biomechanical Phenomena"[MeSH]) OR (biomechanic*[Title/Abstract]) | 182348 |
| 6 | #4 OR #5 | 950459 |
| 7 | ("Cadaver"[Mesh]) OR (cadaver*[Title/Abstract]) | 88030 |
| 8 | #3 AND #6 AND #7 | 238 |

(((((distal radio-ulnar joint[Title/Abstract]) OR (distal radioulnar joint[Title/Abstract])) OR (DRUJ[Title/Abstract])) OR ((("Triangular Fibrocartilage"[MeSH]) OR (triangular fibrocartilage*[Title/Abstract])) OR (TFCC[Title/Abstract]))) AND ((("Anatomy"[MeSH]) OR (Anatom*[Title/Abstract])) OR (("Biomechanical Phenomena"[MeSH]) OR (biomechanic*[Title/Abstract])))) AND (("Cadaver"[Mesh]) OR (cadaver*))

Search EMBASE: 25-01-2022

| 1 | triangular fibrocartilage/ | 760 |
| --- | --- | --- |
| 2 | (distal radio-ulnar joint or distal radioulnar joint or DRUJ or triangular fibrocartilage or TFCC).ti,ab,kw. | 2928 |
| 3 | 1 or 2 | 3059 |
| 4 | exp anatomy/ | 110996 |
| 5 | biomechanics/ | 119302 |
| 6 | (anatom* or biomechanic*).ti,ab,kw. | 578913 |
| 7 | 4 or 5 or 6 | 690100 |
| 8 | cadaver/ | 53967 |
| 9 | cadaver*.ti,ab,kw. | 79551 |
| 10 | 8 or 9 | 93744 |
| 11 | 3 and 7 and 10 | 242 |

*Supplement Table 2. Outcome measure*

P_ET_ = ((Ddis-Drep)/(Ddis-Dint))*100%

P_ET_ = Percentage of eliminated translation.

Ddis = Total translation of the ulna when the TFCC is disrupted.

Drep = Total translation of the ulna when the TFCC is repaired.

Dint = Total translation of ulna when the TFCC is intact, before the disruption.

*Supplement Table 3. Quality assessment*

|  | Objective | Basic information on sample | Methods comprehensibly | condition of specimens | Education of researchers | Findings observed by more than one | Results thoroughly and precise | Statistics appropriate | Details about consistency | Photographs of observations | Discussed within current evidence | Clinical implications | Limitations | Total |
| --- | --- | --- | --- | --- | --- | --- | --- | --- | --- | --- | --- | --- | --- | --- |
| Desai 2013 | Yes | No | Yes | Yes | No | No | Yes | Yes | Yes | Yes | Yes | Yes | Yes | 10  77% |
| Gutiérrez 2021 | Yes | Yes | Yes | No | Yes | Yes | Yes | Yes | No | No | Yes | Yes | Yes | 10  77% |
| Johnson 2019 | Yes | Yes | Yes | No | No | No | Yes | Yes | No | No | Yes | Yes | Yes | 8  62% |
| Ma 2017 | Yes | Yes | Yes | Yes | Yes | No | Yes | Yes | Yes | No | Yes | Yes | Yes | 11  85% |
| Yao 2009 | Yes | No | Yes | No | Yes | No | Yes | Yes | No | No | Yes | Yes | Yes | 8  62% |

*Supplement Table 4. Surgical techniques with suture types for techniques of reinsertion.*

|  | Outside-In Repair | Suture anchor repair | Peripheral Capsular Repair | Transosseous Tunnel Repair |
| --- | --- | --- | --- | --- |
| Desai, 2013 | Two 2-0 PDS sutures, vertical mattress fashion. | 2-0 fiber wires and mini-pushlock suture anchors placed at the fovea. |  |  |
| Gutiérrez, 2021 |  | 2.7 mm Anchor Corkscrew with 2-0 Fiberwire sutures placed at the fovea. |  | Two intra-articular tunnels. Fiberwire 2-0 suture, assisted by a hypodermic needle number 18. |
| Johnson, 2019 |  |  | Three 2-0 PDS sutures, horizontal mattress fashion. | One intra-articular tunnel. Suture lasso, assisted by a 2-0 fiber wire. Secured with a 2-mm Push Lock anchor. |
| Ma, 2017 |  | 3.5 mm suture anchor with ultrabraid sutures placed at the fovea. |  | Two intra-articular tunnels. No. 2 braided USP core suture suture, assisted by a nylon 3-0 suture. |
| Yao, 2009 | Two 2-0 PDS sutures, vertical mattress fashion. |  | FasT-Fix system; two pre-tied 0 ticron sutures. |  |
